# Reward Circuitry and Motivational Deficits in Social Anxiety Disorder: What Can Be Learned From Mouse Models?

**DOI:** 10.3389/fnins.2020.00154

**Published:** 2020-02-26

**Authors:** Corinne N. Carlton, Holly Sullivan-Toole, Merage Ghane, John A. Richey

**Affiliations:** ^1^Clinical Science Program, Department of Psychology, Virginia Tech, Blacksburg, VA, United States; ^2^Graduate Program in Translational Biology, Medicine, and Health, Virginia Tech, Blacksburg, VA, United States

**Keywords:** social anxiety, anhedonia, reward, mouse models, social stress

## Abstract

Social anxiety disorder (SAD) is a common and serious psychiatric condition that typically emerges during adolescence and persists into adulthood if left untreated. Prevailing interventions focus on modulating threat and arousal systems but produce only modest rates of remission. This gap in efficacy suggests that most mainstream treatment concepts do not sufficiently target core processes involved in the onset and maintenance of SAD. This idea has further driven the development of new theoretical models that target dopamine (DA)-driven reward circuitry and motivational deficits that appear to be systematically altered in SAD. Most of the available data linking systemic alterations in DA neurobiology to SAD in humans, although abundant, remains at the level of correlational evidence. Accordingly, the purpose of this brief review is to critically evaluate the relevance of experimental work in rodent models that link details of DA function to symptoms of social anxiety. We conclude that, despite certain systematic limitations inherent in animal models, these approaches provide useful insights into human biomarkers of social anxiety including that (1) adolescence may serve as a critical period for the convergence of neurobiological and environmental factors that modify future expectations about social reward through experience dependent changes in DA-ergic circuitry, (2) females may show unique susceptibility to social anxiety symptoms when encountering relational instability that influences DA-related neural processes, and (3) separate from fear and arousal systems, the functional neurobiology of central DA systems contribute uniquely to susceptibility and maintenance of anhedonic factors relevant to human models of SAD.

## Introduction

Social anxiety disorder (SAD) is a common and debilitating psychiatric disorder predominantly characterized by persistent fear of one or more social or performance situations (American Psychiatric Association, 2013). Prevailing interventions for SAD focus on modulating threat and arousal systems and produce only modest rates of treatment response (Hofmann and Smits, 2008; Cuijpers et al., 2016), with a considerable proportion of individuals (perhaps as high as 60%; Springer et al., 2018) still reporting clinically significant symptoms after completing treatment (Heimberg et al., 1998; Otto et al., 2000). This gap in efficacy has motivated new research aimed at identifying alternative intervention approaches that may enhance clinical outcomes. One potentially promising avenue has identified dysfunction in the mesolimbic dopamine (DA) system, which has been linked to observations of motivational deficits in SAD, and consistent with a social anhedonic phenotype, which has been linked as a correlate of SAD (Tiihonen et al., 1997; Schneier et al., 2000, 2008; Cremers et al., 2015; Richey et al., 2013, 2017, 2019; but see also Schneier et al., 2009), but not other anxiety disorders such as generalized anxiety disorder (Brown et al., 1998). Dysfunction in the ventral striatum (VS) is central to this model, with a number of studies demonstrating reduced activity in the VS in relation to social but not non-social incentives (Richey et al., 2013, 2017; Maresh et al., 2014). Yet, despite the potential promise of this approach, remarkably little is known about the details and neurobiological relevance of DA-ergic neurocircuitry in SAD. Furthermore, because underlying molecular and cellular signaling properties cannot be directly measured or manipulated in humans (excepting rare and invasive instances of surgically implanted microsensors; Kishida et al., 2011, 2016), the available evidence linking systemic alterations in DA neurobiology to social anxiety symptomatology, although abundant, remains correlational. Accordingly, the purposes of this brief review are to (1) describe recent contributions from three commonly used animal models of social stress that induce behavioral phenotypes of anxiety, reduced social interaction, and generalized anhedonia, (2) highlight ways in which social stress alters DA circuitry to drive deficits in motivation for social approach behavior, and (3) critically evaluate their relevance to human models of SAD. In doing so, we highlight both limitations and opportunities to advance extant theory in SAD by considering the implications that follow from experimental manipulations of social processes that cannot be easily or ethically manipulated in humans. The behavioral assays reviewed here are largely agnostic to the experimental manipulations that preceded them (e.g., behavioral, genetic, and pharmacological). However, approach and avoidance motivation in the context of social stressors have also been considered in murine models of neurodevelopmental disorders such as autism (Moy et al., 2004, 2008), although this remains a relatively new area of inquiry and additional work will be required to identify characteristic response patterns in murine models of autism versus anxiety.

## Relevance of Mouse Models of Social Anxiety

*Mus musculus* is a common and highly social mouse species that engages in frequent reciprocal social behavior such as communal nesting, parenting behaviors, territorial scent marking, and aggression (Carter et al., 1992; Miczek et al., 2001; Arakawa et al., 2008). Certain genetic strains of mice, such as c57bl, are more social and sensitive to social reward than other strains (Panksepp and Lahvis, 2007; Panksepp et al., 2007). As such these mouse models are a useful research tool to investigate neurobiological influences on social behavior. Experimental manipulation of naturally occurring parameters of the social environment such as hierarchies, isolation, and defeat leads to profound disruptions in social interaction as well as anxiety-like behavior in mice (Toth and Neumann, 2013). In human research, such manipulations are often not possible due to ethical or feasibility constraints (i.e., ability to experimentally manipulate stress during development, or to manipulate social stress repeatedly and chronically). As such, mouse models can provide a means of examining potential etiological models of SAD.

## Social Defeat Stress Paradigm

The social defeat stress (SDS) paradigm is perhaps the most common social stress paradigm in rodents, and consists of establishing a dominance–subordination relationship between two mice by placing an experimental animal (intruder) into the home cage of a larger and more aggressive conspecific (resident; Golden et al., 2011). The resident typically defends its territory aggressively, consequently subjecting the intruder to social defeat. Following SDS, mice show anxiety-like behaviors including dysregulated ultrasonic vocalizations and alterations in grooming behaviors (Denmark et al., 2010). In addition to these anxiety-related outcomes, SDS had also been linked to the disruption of hedonic processes such as reduced sucrose intake and increased drug self-administration, as well as dysregulation of social behavior, such as reduced social investigation and increased isolation, which have in turn been associated with reduced social interaction in humans (Krishnan et al., 2007; Lukas et al., 2011; Razzoli et al., 2011). Whereas acute SDS does not reliably induce reduced social interaction, chronic SDS results in long-lasting social avoidance across species and across multiple testing paradigms (Avgustinovich et al., 2005; Berton et al., 2006; Krishnan et al., 2007; Venzala et al., 2013). Importantly, while virtually all chronically defeated mice show increased anxiety-like behaviors, only a subset of “anhedonia-susceptible” mice, comprising approximately 50% of those chronically defeated, show a behavioral profile of anhedonia and social avoidance (Krishnan et al., 2007; Huang et al., 2016). This pattern among socially stressed mice suggests that neurobiological vulnerability may give rise to a subgroup within the broader spectrum of human social anxiety, who experience not only anxiety in response to extreme social stressors, but also anhedonic symptoms. However, although the results described above from these specific animal studies may provide clues toward a human model of social anxiety that is specifically influenced by variation in the reward system, they should be interpreted with caution given the potential for species-specific differences and lack of cross-species biological and behavioral markers.

Alterations to the DA system may underlie observed increases in anhedonic behaviors following chronic social defeat. Support for this idea has been provided across a range of studies showing that animals subject to chronic SDS demonstrate increases in DA neuron firing rates and phasic “burst” activity in the ventral tegmental area (VTA), a brain structure heavily implicated in reward processing (Krishnan et al., 2007; Barik et al., 2013; Fernandez et al., 2018). Using optogenetic techniques, Chaudhury et al. (2013) demonstrated that high frequency phasic, but not low frequency tonic optogenetic stimulation of VTA-NAc projecting DA neurons, rapidly increased susceptibility to subthreshold SDS, notably characterized by increased social withdrawal and decreased sucrose preference, whereas this effect was not observed when stimulating VTA-medial prefrontal cortex (mPFC) projecting DA neurons. Interestingly, optical inhibition of VTA-NAc activity promoted resilience to SDS. These causal firing-pattern and circuit-specific findings are consistent with related findings suggesting the involvement of VTA-NAc DA hyperactivity in SDS-induced social avoidance using *ex vivo* and *in vivo* measurement techniques (Krishnan et al., 2007; Cao et al., 2010). Susceptible animals also show reduced neuronal firing from vmPFC, and given that vmPFC-VTA projections are important in the fine tuning (i.e., increasing signal to noise ratio) of DA activity coming from VTA to NAc, this suggests an important role in this circuit for maintaining goal-directed behavior, rather than promoting habitual responses that are driven by other limbic regions (Patton et al., 2013; Abe et al., 2019). These findings further suggest that reduced regulation of DA activity due to reduced vmPFC-VTA and increased VTA-NAc activity may be a part of reduced motivational salience associated with rewarding stimuli that is observed in SDS susceptible rodents. Together, these factors highlight that reduced vmPFC regulation of DA activity in VTA may increase susceptibility to SDS by increasing phasic DA firing along the VTA-NAc circuit during experience with chronic SDS. The consequences of this may include reduced attention to potentially rewarding stimuli (e.g., Pessoa, 2015) and/or reductions in expected value derived from reward-seeking behaviors (e.g., Schultz et al., 2017).

Socially avoidant behaviors stemming from social defeat also appear to generalize across various environmental contexts, suggesting that responses to social defeat may be observed in situations unrelated to prior SDS experiences. For example, mice who witnessed the social defeat of another mouse spend significantly less time interacting with other mice, and demonstrate reduced time engaging in social behaviors with conspecifics (Iñiguez et al., 2014; Sial et al., 2016). Further, these reductions in social behaviors appear to persist into adulthood in mice who experienced SDS during adolescence, and become more persistent as development progresses (Zhang et al., 2016; Mouri et al., 2018), suggesting an overall withdrawal from social contact that generalizes to future contexts. Moreover, following SDS during adolescence, rodents show increased DA release in the mPFC, with DA turnover levels remaining elevated long after the stressor is removed, before falling below normal levels (Watt et al., 2014). Interestingly, this long-term DA hypofunction in the mPFC results only from SDS exposure during adolescence, indicating that the encoding of stress on DA-circuitry may be profoundly impacted by developmental factors. Even in adulthood, however, there is evidence that social stress can result in a susceptible phenotype. For example, a recent study by De Miguel et al. (2019) reported that adult mice exposed to SDS demonstrated increased neurogenesis in areas of the hippocampus specifically associated with new memory formation, and that these neurons were particularly sensitized to subsequent social stressors. Further, a study by Lagace et al. (2010) also demonstrated increased hippocampal neurogenesis following SDS in adult mice, with stress-born neurons surviving primarily in mice who had displayed persistent social avoidance post-social defeat. Together, these results suggest that social stress may predispose susceptible animals to further stress vulnerability via increased hippocampal responsivity to social stressors.

These findings collectively highlight both the general utility of the SDS paradigm in interrogating social stress–reward interactions in mice as well as the potential to inform developmental theories of SAD in humans (e.g., Richey et al., 2019). The particularly pronounced and longer-lasting effects on DA circuitry following the experience of SDS during adolescence may specifically relate to epidemiologic findings in humans that suggest a potentially sensitive period for SAD development (i.e., a median age of onset at or around 13 years of age; Wittchen et al., 1999; Gregory et al., 2007). Thus, a key point that emerges when considering the factors revealed by SDS in mice is the importance of the adolescent period where early social stress leads to reductions in vmPFC regulation of baseline DA activity in the VTA. This has a long-term effect of reinforcing habitual stimulus–response patterns stemming from overactive VTA-NAc circuits during future experiences with stress at the expense of goal-oriented behavior that may increase chances of detecting or experiencing reward.

## Social Instability Paradigm

Social instability paradigms involve a complete disruption of social hierarchies within an animal’s environment by randomly changing the cage composition of residents (Haller et al., 1999). This reduces overall environmental predictability and the ability to form reliable expectations about the outcome of social interactions. Disruptions in reward-related behaviors are among the most commonly observed outcomes in this paradigm, along with HPA axis dysfunction and increased anxiety-like behaviors (Goñi-Balentziaga et al., 2018). For example, Schmidt et al. (2010) demonstrated that following a period of social instability, mice exhibit behaviors consistent with both anxiety and reduced social interaction, including decreases in general and social investigation, increased head dips (associated with anxiety) during elevated-plus maze tasks, as well as reduced initial investigation during open-field tasks (Sterlemann et al., 2008; Lu et al., 2019). Like the SDS paradigm, long-lasting effects have been reported for social instability when this paradigm is performed in adolescence, and these effects may also be transmitted to future offspring. For example, Saavedra-Rodríguez and Feig (2012) demonstrated that enhanced anxiety-like behaviors and social deficits in offspring of stressed parents were not due to alterations in postnatal parental behavior, but instead to offspring’s enhanced expression of genes associated with synaptic plasticity.

The social instability paradigm, like SDS, has not only been linked to anxiety-like behaviors and reduced social interaction, but also to a more generalized form of anhedonia. For example, this paradigm increases rates of avoidance across general novel stimuli, which includes, but is not limited to, avoidance of social interaction (Saavedra-Rodríguez and Feig, 2012). Additionally, stressed mice show reduced saccharin consumption reflecting reduced reward motivation and attenuated DA release in the nucleus accumbens (NAc) but no difference in tonic DA levels following cocaine injection (Shimamoto et al., 2011). This finding suggests that animals who experienced chronic social instability may show reduced reinforcement from typically rewarding stimuli (e.g., saccharin, cocaine), specifically via reduced phasic DA release.

Although research comparing the effects of social instability to other forms of stress is relatively sparse, a number of investigations have revealed that the effects of social instability stress in particular may be sex-specific. For example, an early study by Haller et al. (1999) demonstrated that social instability may be more stressful for females than males as evidenced by sex- and paradigm-specific differences in weight gain and adrenal/plasma corticosterone levels. Recent investigations in mice have further reinforced the idea that social instability is sufficient to produce sustained but reversible (upon return to stable housing) HPA axis activation in female mice (Jarcho et al., 2016). Further, Labaka et al. (2017) reported that female mice exposed to social instability stress showed changes in central DA-ergic activity including reductions in hippocampal DA concentrations, which has been theoretically associated with stress-linked impairments in memory for positive material associated with DA deficiency and anhedonia in humans (Dillon and Pizzagalli, 2018). Moreover, social instability in females has been shown to produce anhedonia without influencing novelty-seeking behavior or hormone levels, whereas the reverse was the case for chronic mild non-social stress (e.g., food deprivation, restraint stress, white noise; Dadomo et al., 2018).

Along similar lines, gender differences in humans with SAD have long been acknowledged [see Asher et al. (2017) for an integrative review], and the motivational consequences of social instability may relate to several observations of gender-specific relational effects in human samples. Epidemiological work has demonstrated that SAD is more prevalent in females (lifetime prevalence: 5.67%) compared to males (4.2%; Xu et al., 2012; Asher and Aderka, 2018) and females with SAD are significantly more likely to report major depression (which comprises a significant anhedonic component) than men (Beesdo et al., 2007; Xu et al., 2012; although see Asher and Aderka, 2018). Additionally, higher levels of relational interdependence in females have been positively associated with the severity of social anxiety symptoms, perhaps reflecting a susceptible context for social instability that would otherwise be buffered at higher levels of social independence (Moscovitch et al., 2005). Thus, although specific neurobiological linkages between social instability, central DA function, and reward remain in its infancy, the animal work reviewed here suggests that the motivational consequences of relational instability may be related to central DA function and is particularly pronounced in females. It should be noted that although the social instability model may produce seemingly analogous links to human social anxiety symptomology, due to the subjective nature of symptoms and lack of established biomarkers for the disorder, no complete conclusions can be drawn. However, invaluable clues can be gleaned from these models about the function, and variation in function, in these highly conserved dopaminergic systems.

## Social Isolation Paradigm

Social isolation paradigms involve the separation of experimental animals into individual housing during weaning until adulthood. Isolation functionally prevents normative social interaction over the course of development, ultimately producing anxiety-like behaviors that appear to be socially specific, such as avoidance of conspecifics, increased ultrasonic vocalizations (i.e., calls of distress to other conspecifics), and reduced approach-related behaviors to novel mice (Huang et al., 2017). However, social isolation has also been shown to increase general anxiety-like behaviors such as a novelty-induced avoidance, decreased environmental exploration, and reduced time spent in vulnerable positions (e.g., open portions of a plus maze; Barnes et al., 2017; Huang et al., 2017; Caruso et al., 2018). Further, socially isolated mice have been shown to display higher locomotor activity, longer rates of immobility in tail suspension tests, increased adipose mass, and increased voluntary ethanol consumption later in life (Lopez et al., 2011; Sun et al., 2014; Lopez and Laber, 2015; Ieraci et al., 2016). The DA system is significantly impacted by this paradigm, such that following social isolation, mice have demonstrated increased DA-receptor 2 mRNA and protein levels in the NAc, suggestive of excessive dopaminergic activity in mesolimbic areas that are key to motivation and reward salience (Li et al., 2017). Additionally, following social isolation in adolescence, mice show increases in mesolimbic DA-ergic activation in response to cocaine in adulthood, thereby suggesting a long-term impact of social stress on reward sensitivity that persists throughout development (Fosnocht et al., 2019).

This paradigm similarly may have translational relevance for human isolative behaviors. While only one study in humans has specifically identified social isolation as a risk factor (Hayward et al., 1998), other studies have identified that isolative behaviors of parents (e.g., insulating children and adolescents from routine social experiences, excessive concern over other’s opinions) may play a causal role in social anxiety development among youth (Leung et al., 1994; Caster et al., 1999). Whereas results from the other animal paradigms reviewed here (SDS and social instability) seem to more directly parallel observations in humans of elevated social anxiety symptoms in chaotic/abusive home environments and/or bullying, social isolation during adolescence may be a relevant model for characterizing social deficits that develop in the context of early adversity during adolescence and are perpetuated by disorder-related social avoidance. Given that experimental evidence in mice (e.g., Fosnocht et al., 2019) illustrates that isolation during adolescence can result in altered reward responsiveness, suggesting that teens who are socially isolated, even in the absence of other social stressors, may experience long-lasting anhedonic symptoms mediated by altering the responsiveness of reward-related brain circuitry. Again, this should be considered with caution, as these animal models can only provide clues toward these potential risk-factors that induce altered reward responsiveness given that a mouse model will never fully capture the complexity of the environmental and social phenomena within the human experience.

## Conclusion: Overall Relevance of Animal Social Stress Paradigms to Human Models of Sad

In summary, this review draws three broad human-relevant conclusions from experimental manipulations of the social environment in mice: (1) adolescence is likely a critical period for the convergence of neurobiological sensitivity with environmental factors to entrench future expectations about social reward through experience dependent modification of DA-ergic circuitry, (2) females may show unique susceptibility to social anxiety symptoms when encountering instability of relational social hierarchies mediated by DA-related neural processes, and (3) separate from fear and arousal systems, the functional neurobiology of central DA systems contribute uniquely to susceptibility and maintenance of anhedonic factors relevant to providing clues for human models of SAD, thus warranting the investigation of new treatment avenues specifically related to the modification of these systems.

Seminal theories of SAD have been largely couched in cognitive and behavioral terms that presuppose causal roles for the uniquely human capacity for propositional and abstract thought (e.g., Clark and Wells, 1995; Hofmann, 2007), particularly as they relate to fear- and threat-focused cognitions regarding future negative evaluation (Hope et al., 1990). However, the proliferation of rodent models of social anxiety and their plausible conceptual linkages to distinctive facets of the human SAD phenotype force at least some reconsideration of the comparative neurobiology involved in core social anxiety processes. Additionally, there are inherent limitations to inferring causal mechanisms from purely behavioral-level observations because social avoidance can be due to dysfunction either within negative valence systems (e.g., social anxiety) or within positive valence systems (e.g., social anhedonia). Thus, little is currently known about how these emotion systems and their respective neural circuits contribute to SAD-like behavioral phenotypes. Further, the presence of a social deficit merely coinciding with an indication of general anhedonia (e.g., reduced saccharin preference) cannot alone pinpoint the source of diminished social interaction as attributable to social anhedonia (with a primary deficit in reward sensitivity) as opposed to social anxiety (with a primary deficit in flight or fight responsivity). While many of these questions remain unanswered, murine models are ideal for dissociating the distinctive influences of positive and negative valence systems on social dysfunction relevant to SAD. Accordingly, the focus of this brief review was on motivational and hedonic deficits linked to comparative mammalian neurobiology, which are increasingly recognized as a relevant aspect of SAD in humans. It should be noted that although these experimental models have credible ties to certain aspects of the SAD phenotype in humans, it is unlikely that any animal model will ever recapitulate all aspects of psychiatric disorders observed in humans (Markou et al., 2009; Nestler and Hyman, 2010; McGonigle and Ruggeri, 2014). Further, Nestler and Hyman (2010) note that for virtually all DSM-defined disorders, there are no verified molecular or cellular abnormalities in human neuropsychiatric disorders that could validate these phenotypes in any animal, meaning that the field lacks anchor-points even for establishing correspondence between *in vivo* disease systems across species. On the one hand, these and related criticisms continue to drive vigorous debate regarding the relevance of animal models to the study of human neuropsychiatric disorders (e.g., Stewart et al., 2014; Anderzhanova et al., 2017; Kafkafi et al., 2018). On the other hand, selected domains of psychiatry have disproportionately benefited from molecular and systems neuroscience work in non-human vertebrates (e.g., Stewart et al., 2012; Treadway and Zald, 2013; Coleman and Pierre, 2014), thus future animal work at the intersection of social anxiety and anhedonia is likely to reveal actionable targets that can be engaged therapeutically in humans.

## Author Contributions

CC, HS-T, MG, and JR were involved in the conception, drafting, and revisions of the manuscript.

## Conflict of Interest

The authors declare that the research was conducted in the absence of any commercial or financial relationships that could be construed as a potential conflict of interest.

## References

[B1] Abe R. Okada S. Nakayama R. Ikegaya Y. Sasaki T. (2019). Social defeat stress causes selective attenuation of neuronal activity in the ventromedial prefrontal cortex. *Sci. Rep.* 9:9447. 10.1038/s41598-019-45833-5 31263153 PMC6603183

[B2] American Psychiatric Association (2013). *Diagnostic and Statistical Manual of Mental Disorders*, 5th Edn Arlington, VA: American Psychiatric Association.

[B3] Anderzhanova E. Kirmeier T. Wotjak C. T. (2017). Animal models in psychiatric research: the RDoC system as a new framework for endophenotype-oriented translational neuroscience. *Neurobiol. Stress* 7 47–56. 10.1016/j.ynstr.2017.03.003 28377991 PMC5377486

[B4] Arakawa H. Blanchard D. C. Arakawa K. Dunlap C. Blanchard R. J. (2008). Scent marking behavior as an odorant communication in mice. *Neurosci. Biobehav. Rev.* 32 1236–1248. 10.1371/journal.pone.0020631 18565582 PMC2577770

[B5] Asher M. Aderka I. M. (2018). Gender differences in social anxiety disorder. *J. Clin. Psychol.* 74 1730–1741. 10.1002/jclp.22624 29667715

[B6] Asher M. Asnaani A. Aderka I. M. (2017). Gender differences in social anxiety disorder: a review. *Clin. Psychol. Rev.* 56 1–12. 10.1016/j.cpr.2017.05.004 28578248

[B7] Avgustinovich D. F. Kovalenko I. L. Kudryavtseva N. N. (2005). A model of anxious depression: persistence of behavioral pathology. *Neurosci. Behav. Physiol.* 35 917–924. 10.1007/s11055-005-0146-6 16270173

[B8] Barik J. Marti F. Morel C. Fernandez S. P. Lanteri C. Godeheu G. (2013). Chronic stress triggers social aversion via glucocorticoid receptor in dopaminoceptive neurons. *Science* 339 332–335. 10.1126/science.1226767 23329050

[B9] Barnes T. D. Rieger M. A. Dougherty J. D. Holy T. E. (2017). Group and individual variability in mouse pup isolation calls recorded on the same day show stability. *Front. Behav. Neurosci.* 11:243. 10.3389/fnbeh.2017.00243 29326565 PMC5736564

[B10] Beesdo K. Bittner A. Pine D. S. Stein M. B. Höfler M. Lieb R. (2007). Incidence of social anxiety disorder and the consistent risk for secondary depression in the first three decades of life. *Arch. Gen. Psychiatry* 64 903–912. 17679635 10.1001/archpsyc.64.8.903

[B11] Berton O. McClung C. A. DiLeone R. J. Krishnan V. Renthal W. Russo S. J. (2006). Essential role of BDNF in the mesolimbic dopamine pathway in social defeat stress. *Science* 311 864–868. 10.1126/science.1120972 16469931

[B12] Brown T. A. Chorpita B. F. Barlow D. H. (1998). Structural relationships among dimensions of the DSM-IV anxiety and mood disorders and dimensions of negative affect, positive affect, and autonomic arousal. *J. Abnorm. Psychol.* 107 179–192. 10.1037/0021-843x.107.2.179 9604548

[B13] Cao J. L. Covington H. E. Friedman A. K. Wilkinson M. B. Walsh J. J. Cooper D. (2010). Mesolimbic dopamine neurons in the brain reward circuit mediate susceptibility to social defeat and antidepressant action. *J. Neurosci.* 30 16453–16458. 10.1523/JNEUROSCI.3177-10.2010 21147984 PMC3061337

[B14] Carter C. S. Williams J. R. Witt D. M. Insel T. R. (1992). Oxytocin and social bonding A. *Ann. N. Y. Acad. Sci.* 652 204–211. 1626829 10.1111/j.1749-6632.1992.tb34356.x

[B15] Caruso M. J. Crowley N. A. Reiss D. E. Caulfield J. I. Luscher B. Cavigelli S. A. (2018). Adolescent social stress increases anxiety-like behavior and alters synaptic transmission, without influencing nicotine responses, in a sex-dependent manner. *Neuroscience* 373 182–198. 10.1016/j.neuroscience.2018.01.006 29343455 PMC5816715

[B16] Caster J. B. Inderbitzen H. M. Hope D. (1999). Relationship between youth and parent perceptions of family environment and social anxiety. *J. Anxiety Disord.* 13 237–251. 10.1016/s0887-6185(99)00002-x 10372340

[B17] Chaudhury D. Walsh J. J. Friedman A. K. Juarez B. Ku S. M. Koo J. W. (2013). Rapid regulation of depression-related behaviours by control of midbrain dopamine neurons. *Nature* 493 532–536. 10.1038/nature11713 23235832 PMC3554860

[B18] Clark D. M. Wells A. (1995). “A cognitive model of social phobia,” in *Social phobia: Diagnosis, assessment, and treatment*, eds Heimberg R. G. Liebowitz M. R. Hope D. A. Schneier F. R. (New York, NY: The Guilford Press), 69–93.

[B19] Coleman K. Pierre P. J. (2014). Assessing anxiety in nonhuman primates. *ILAR J.* 55 333–346. 10.1093/ilar/ilu019 25225310 PMC4240439

[B20] Cremers H. R. Veer I. M. Spinhoven P. Rombouts S. A. Roelofs K. (2015). Neural sensitivity to social reward and punishment anticipation in social anxiety disorder. *Front. Behav. Neurosci.* 8:439. 10.3389/fnbeh.2014.00439 25601830 PMC4283602

[B21] Cuijpers P. Cristea I. A. Karyotaki E. Reijnders M. Huibers M. J. (2016). How effective are cognitive behavior therapies for major depression and anxiety disorders? A meta-analytic update of the evidence. *World Psychiatry* 15 245–258. 10.1002/wps.20346 27717254 PMC5032489

[B22] Dadomo H. Gioiosa L. Cigalotti J. Ceresini G. Parmigiani S. Palanza P. (2018). What is stressful for females? Differential effects of unpredictable environmental or social stress in CD1 female mice. *Horm. Behav.* 98 22–32. 10.1016/j.yhbeh.2017.11.013 29187314

[B23] De Miguel Z. Haditsch U. Palmer T. D. Azpiroz A. Sapolsky R. M. (2019). Adult-generated neurons born during chronic social stress are uniquely adapted to respond to subsequent chronic social stress. *Mol. Psychiatry* 24 1178–1188. 10.1038/s41380-017-0013-1 29311652

[B24] Denmark A. Tien D. Wong K. Chung A. Cachat J. Goodspeed J. (2010). The effects of chronic social defeat stress on mouse self-grooming behavior and its patterning. *Behav. Brain Res.* 208 553–559. 10.1016/j.bbr.2009.12.041 20060021

[B25] Dillon D. G. Pizzagalli D. A. (2018). Mechanisms of memory disruption in depression. *Trends Neurosci.* 41 137–149. 10.1016/j.tins.2017.12.006 29331265 PMC5835184

[B26] Fernandez S. P. Broussot L. Marti F. Contesse T. Mouska X. Soiza-Reilly M. (2018). Mesopontine cholinergic inputs to midbrain dopamine neurons drive stress-induced depressive-like behaviors. *Nat. Commun.* 9:4449. 10.1038/s41467-018-06809-7 30361503 PMC6202358

[B27] Fosnocht A. Q. Lucerne K. E. Ellis A. S. Olimpo N. A. Briand L. A. (2019). Adolescent social isolation increases cocaine seeking in male and female mice. *Behav. Brain Res.* 359 589–596. 10.1016/j.bbr.2018.10.007 30296530 PMC6557448

[B28] Golden S. A. Covington H. E. III Berton O. Russo S. J. (2011). A standardized protocol for repeated social defeat stress in mice. *Nat. Protoc.* 6 1183–1191. 10.1038/nprot.2011.361 21799487 PMC3220278

[B29] Goñi-Balentziaga O. Perez-Tejada J. Renteria-Dominguez A. Lebena A. Labaka A. (2018). Social instability in female rodents as a model of stress related disorders: a systematic review. *Physiol. Behav.* 196 190–199. 10.1016/j.physbeh.2018.09.001 30196085

[B30] Gregory A. M. Caspi A. Moffitt T. E. Koenen K. Eley T. C. Poulton R. (2007). Juvenile mental health histories of adults with anxiety disorders. *Am. J. Psychiatry* 164 301–308. 10.1176/ajp.2007.164.2.301 17267794

[B31] Haller J. Fuchs E. Halász J. Makara G. B. (1999). Defeat is a major stressor in males while social instability is stressful mainly in females: towards the development of a social stress model in female rats. *Brain Res. Bull.* 50 33–39. 10.1016/s0361-9230(99)00087-8 10507469

[B32] Hayward C. Killen J. D. Kraemer H. C. Taylor C. B. (1998). Linking self-reported childhood behavioral inhibition to adolescent social phobia. *J. Am. Acad. Child Adolesc. Psychiatry* 37 1308–1316. 10.1097/00004583-199812000-00015 9847504

[B33] Heimberg R. G. Liebowitz M. R. Hope D. A. Schneier F. R. Holt C. S. Welkowitz L. A. (1998). Cognitive behavioral group therapy vs phenelzine therapy for social phobia: 12-week outcome. *Arch. Gen. Psychiatry* 55 1133–1141. 9862558 10.1001/archpsyc.55.12.1133

[B34] Hofmann S. G. (2007). Cognitive factors that maintain social anxiety disorder: a comprehensive model and its treatment implications. *Cogn. Behav. Ther.* 36 193–209. 10.1080/16506070701421313 18049945 PMC2151931

[B35] Hofmann S. G. Smits J. A. (2008). Cognitive-behavioral therapy for adult anxiety disorders: a meta-analysis of randomized placebo-controlled trials. *J. Clin. Psychiatry* 69 621–32. 18363421 10.4088/jcp.v69n0415 PMC2409267

[B36] Hope D. A. Rapee R. M. Heimberg R. G. Dombeck M. J. (1990). Representations of the self in social phobia: vulnerability to social threat. *Cogn. Ther. Res.* 14 177–189. 10.1007/bf01176208

[B37] Huang G.-B. Zhao T. Gao X.-L. Zhang H.-X. Xu Y.-M. Li H. (2016). Effect of chronic social defeat stress on behaviors and dopamine receptor in adult mice. *Prog. Neuropsychopharmacol. Biol. Psychiatry* 66 73–79. 10.1016/j.pnpbp.2015.12.002 26655446

[B38] Huang Q. Zhou Y. Liu L. Y. (2017). Effect of post-weaning isolation on anxiety-and depressive-like behaviors of C57BL/6J mice. *Exp. Brain Res.* 235 2893–2899. 10.1007/s00221-017-5021-5 28695280

[B39] Ieraci A. Mallei A. Popoli M. (2016). Social isolation stress induces anxious-depressive-like behavior and alterations of neuroplasticity-related genes in adult male mice. *Neural Plast.* 2016:6212983. 10.1155/2016/6212983 26881124 PMC4736811

[B40] Iñiguez S. D. Riggs L. M. Nieto S. J. Dayrit G. Zamora N. N. Shawhan K. L. (2014). Social defeat stress induces a depression-like phenotype in adolescent male c57BL/6 mice. *Stress* 17 247–255. 10.3109/10253890.2014.910650 24689732 PMC5534169

[B41] Jarcho M. R. Massner K. J. Eggert A. R. Wichelt E. L. (2016). Behavioral and physiological response to onset and termination of social instability in female mice. *Horm. Behav.* 78 135–140. 10.1016/j.yhbeh.2015.11.004 26597994

[B42] Kafkafi N. Agassi J. Chesler E. J. Crabbe J. C. Crusio W. E. Eilam D. (2018). Reproducibility and replicability of rodent phenotyping in preclinical studies. *Neurosci. Biobehav. Rev.* 87 218–232. 10.1016/j.neubiorev.2018.01.003 29357292 PMC6071910

[B43] Kishida K. T. Saez I. Lohrenz T. Witcher M. R. Laxton A. W. Tatter S. B. (2016). Subsecond dopamine fluctuations in human striatum encode superposed error signals about actual and counterfactual reward. *Proc. Natl. Acad. Sci. U.S.A.* 113 200–205. 10.1073/pnas.1513619112 26598677 PMC4711839

[B44] Kishida K. T. Sandberg S. G. Lohrenz T. Comair Y. G. Sáez I. Phillips P. E. (2011). Sub-second dopamine detection in human striatum. *PLoS One* 6:e23291. 10.1371/journal.pone.0023291 21829726 PMC3150430

[B45] Krishnan V. Han M. H. Graham D. L. Berton O. Renthal W. Russo S. J. (2007). Molecular adaptations underlying susceptibility and resistance to social defeat in brain reward regions. *Cell* 131 391–404. 10.1016/j.cell.2007.09.018 17956738

[B46] Labaka A. Gómez-Lázaro E. Vegas O. Pérez-Tejada J. Arregi A. Garmendia L. (2017). Reduced hippocampal IL-10 expression, altered monoaminergic activity and anxiety and depressive-like behavior in female mice subjected to chronic social instability stress. *Behav. Brain Res.* 335 8–18. 10.1016/j.bbr.2017.08.002 28789949

[B47] Lagace D. C. Donovan M. H. DeCarolis N. A. Farnbauch L. A. Malhotra S. Berton O. (2010). Adult hippocampal neurogenesis is functionally important for stress-induced social avoidance. *Proc. Natl. Acad. Sci. U.S.A.* 107 4436–4441. 10.1073/pnas.0910072107 20176946 PMC2840117

[B48] Leung A. W. Heimberg R. G. Holt C. S. Bruch M. A. (1994). Social anxiety and perception of early parenting among American, Chinese American, and social phobic samples. *Anxiety* 1 80–89. 10.1002/anxi.3070010207 9160552

[B49] Li B. J. Liu P. Chu Z. Shang Y. Huan M. X. Dang Y. H. (2017). Social isolation induces schizophrenia-like behavior potentially associated with HINT1, NMDA receptor 1, and dopamine receptor 2. *Neuroreport* 28 462–469. 10.1097/WNR.0000000000000775 28410269 PMC5639997

[B50] Lopez M. F. Doremus-Fitzwater T. L. Becker H. C. (2011). Chronic social isolation and chronic variable stress during early development induce later elevated ethanol intake in adult C57BL/6J mice. *Alcohol* 45 355–364. 10.1016/j.alcohol.2010.08.017 20880662 PMC3013234

[B51] Lopez M. F. Laber K. (2015). Impact of social isolation and enriched environment during adolescence on voluntary ethanol intake and anxiety in C57BL/6J mice. *Physiol. Behav.* 148 151–156. 10.1016/j.physbeh.2014.11.012 25446196 PMC4425642

[B52] Lu Q. Mouri A. Yang Y. Kunisawa K. Teshigawara T. Hirakawa M. (2019). Chronic unpredictable mild stress-induced behavioral changes are coupled with dopaminergic hyperfunction and serotonergic hypofunction in mouse models of depression. *Behav. Brain Res.* 372:112053. 10.1016/j.bbr.2019.112053 31288060

[B53] Lukas M. Toth I. Reber S. O. Slattery D. A. Veenema A. H. Neumann I. D. (2011). The neuropeptide oxytocin facilitates pro-social behavior and prevents social avoidance in rats and mice. *Neuropsychopharmacology* 36 2159–2168. 10.1038/npp.2011.95 21677650 PMC3176581

[B54] Maresh E. L. Allen J. P. Coan J. A. (2014). Increased default mode network activity in socially anxious individuals during reward processing. *Biol. Mood Anxiety Disord.* 4:7. 10.1186/2045-5380-4-7 25075275 PMC4114426

[B55] Markou A. Chiamulera C. Geyer M. A. Tricklebank M. Steckler T. (2009). Removing obstacles in neuroscience drug discovery: the future path for animal models. *Neuropsychopharmacology* 34 74–89. 10.1038/npp.2008.173 18830240 PMC2651739

[B56] McGonigle P. Ruggeri B. (2014). Animal models of human disease: challenges in enabling translation. *Biochem. Pharmacol.* 87 162–171. 10.1016/j.bcp.2013.08.006 23954708

[B57] Miczek K. A. Maxson S. C. Fish E. W. Faccidomo S. (2001). Aggressive behavioral phenotypes in mice. *Behav. Brain Res.* 125 167–181. 10.1016/s0166-4328(01)00298-4 11682108

[B58] Moscovitch D. A. Hofmann S. G. Litz B. T. (2005). The impact of self-construals on social anxiety: a gender-specific interaction. *Pers. Individ. Dif.* 38 659–672. 10.1016/j.paid.2004.05.021

[B59] Mouri A. Ukai M. Uchida M. Hasegawa S. Taniguchi M. Ito T. (2018). Juvenile social defeat stress exposure persistently impairs social behaviors and neurogenesis. *Neuropharmacology* 133 23–37. 10.1016/j.neuropharm.2018.01.016 29337227

[B60] Moy S. S. Nadler J. J. Perez A. Barbaro R. P. Johns J. M. Magnuson T. R. (2004). Sociability and preference for social novelty in five inbred strains: an approach to assess autistic-like behavior in mice. *Genes Brain Behav.* 3 287–302. 10.1111/j.1601-1848.2004.00076.x 15344922

[B61] Moy S. S. Nadler J. J. Young N. B. Nonneman R. J. Segall S. K. Andrade G. M. (2008). Social approach and repetitive behavior in eleven inbred mouse strains. *Behav. Brain Res.* 191 118–129. 10.1016/j.bbr.2008.03.015 18440079 PMC2441761

[B62] Nestler E. J. Hyman S. E. (2010). Animal models of neuropsychiatric disorders. *Nat. Neurosci.* 13 1161–1169. 10.1038/nn.2647 20877280 PMC3750731

[B63] Otto M. W. Pollack M. H. Gould R. A. Worthington J. J. III McArdle E. T. Rosenbaum J. F. (2000). A comparison of the efficacy of clonazepam and cognitive-behavioral group therapy for the treatment of social phobia. *J. Anxiety Disord.* 14 345–358. 10.1016/s0887-6185(00)00027-x 11043885

[B64] Panksepp J. B. Jochman K. A. Kim J. U. Koy J. J. Wilson E. D. Chen Q. (2007). Affiliative behavior, ultrasonic communication and social reward are influenced by genetic variation in adolescent mice. *PLoS One* 2:e351. 10.1371/journal.pone.0000351 17406675 PMC1831495

[B65] Panksepp J. B. Lahvis G. P. (2007). Social reward among juvenile mice. *Genes Brain Behav.* 6 661–671. 10.1111/j.1601-183x.2006.00295.x 17212648 PMC2040181

[B66] Patton M. H. Bizup B. T. Grace A. A. (2013). The infralimbic cortex bidirectionally modulates mesolimbic dopamine neuron activity via distinct neural pathways. *J. Neurosci.* 33 16865–16873. 10.1523/JNEUROSCI.2449-13.2013 24155293 PMC3807020

[B67] Pessoa L. (2015). Multiple influences of reward on perception and attention. *Vis. Cogn.* 23 272–290. 10.1080/13506285.2014.974729 26190929 PMC4503337

[B68] Razzoli M. Carboni L. Andreoli M. Ballottari A. Arban R. (2011). Different susceptibility to social defeat stress of BalbC and C57BL6/J mice. *Behav. Brain Res.* 216 100–108. 10.1016/j.bbr.2010.07.014 20654656

[B69] Richey J. Ghane M. Valdespino A. Coffman M. C. Strege M. V. White S. W. (2017). Spatiotemporal dissociation of brain activity underlying threat and reward in social anxiety disorder. *Soc. Cogn. Affect. Neurosci.* 12 81–94. 10.1093/scan/nsw149 27798252 PMC5390704

[B70] Richey J. A. Brewer J. A. Sullivan-Toole H. Strege M. V. Kim-Spoon J. White S. W. (2019). Sensitivity shift theory: a developmental model of positive affect and motivational deficits in social anxiety disorder. *Clin. Psychol. Rev.* 72:101756. 10.1016/j.cpr.2019.101756 31351312

[B71] Richey J. A. Rittenberg A. Hughes L. Damiano C. R. Sabatino A. Miller S. (2013). Common and distinct neural features of social and non-social reward processing in autism and social anxiety disorder. *Soc. Cogn. Affect. Neurosci.* 9 367–377. 10.1093/scan/nss146 23223206 PMC3980795

[B72] Saavedra-Rodríguez L. Feig L. A. (2012). Chronic social instability induces anxiety and defective social interactions across generations. *Biol. Psychiatry* 73 44–53. 10.1016/j.biopsych.2012.06.035 22906514 PMC3826464

[B73] Schmidt M. V. Scharf S. H. Liebl C. Harbich D. Mayer B. Holsboer F. (2010). A novel chronic social stress paradigm in female mice. *Horm. Behav.* 57 415–420. 10.1016/j.yhbeh.2010.01.010 20100488

[B74] Schneier F. R. Abi-Dargham A. Martinez D. Slifstein M. Hwang D. R. Liebowitz M. R. (2009). Dopamine transporters, D_2_ receptors, and dopamine release in generalized social anxiety disorder. *Depress. Anxiety* 26 411–418. 10.1002/da.20543 19180583 PMC2679094

[B75] Schneier F. R. Liebowitz M. R. Abi-Dargham A. Zea-Ponce Y. Lin S. H. Laruelle M. (2000). Low dopamine D_2_ receptor binding potential in social phobia. *Am. J. Psychiatry* 157 457–459. 10.1176/appi.ajp.157.3.457 10698826

[B76] Schneier F. R. Martinez D. Abi-Dargham A. Zea-Ponce Y. Simpson H. B. Liebowitz M. R. (2008). Striatal dopamine D_2_ receptor availability in OCD with and without comorbid social anxiety disorder: preliminary findings. *Depress. Anxiety* 25 1–7. 10.1002/da.20268 17252580

[B77] Schultz W. Stauffer W. R. Lak A. (2017). The phasic dopamine signal maturing: from reward via behavioural activation to formal economic utility. *Curr. Opin. Neurobiol.* 43 139–148. 10.1016/j.conb.2017.03.013 28390863

[B78] Shimamoto A. DeBold J. F. Holly E. N. Miczek K. A. (2011). Blunted accumbal dopamine response to cocaine following chronic social stress in female rats: exploring a link between depression and drug abuse. *Psychopharmacology* 218 271–279. 10.1007/s00213-011-2364-7 21638221 PMC3901580

[B79] Sial O. K. Warren B. L. Alcantara L. F. Parise E. M. Bolaños-Guzmán C. A. (2016). Vicarious social defeat stress: bridging the gap between physical and emotional stress. *J. Neurosci. Methods* 258 94–103. 10.1016/j.jneumeth.2015.10.012 26545443 PMC4691556

[B80] Springer K. S. Levy H. C. Tolin D. F. (2018). Remission in CBT for adult anxiety disorders: a meta-analysis. *Clin. Psychol. Rev.* 61 1–8. 10.1016/j.cpr.2018.03.002 29576326

[B81] Sterlemann V. Ganea K. Liebl C. Harbich D. Alam S. Holsboer F. (2008). Long-term behavioral and neuroendocrine alterations following chronic social stress in mice: implications for stress-related disorders. *Horm. Behav.* 53 386–394. 10.1016/j.yhbeh.2007.11.001 18096163

[B82] Stewart A. Gaikwad S. Kyzar E. Green J. Roth A. Kalueff A. V. (2012). Modeling anxiety using adult zebrafish: a conceptual review. *Neuropharmacology* 62 135–143. 10.1016/j.neuropharm.2011.07.037 21843537 PMC3195883

[B83] Stewart A. M. Nguyen M. Wong K. Poudel M. K. Kalueff A. V. (2014). Developing zebrafish models of autism spectrum disorder (ASD). *Prog. Neuropsychopharmacol. Biol. Psychiatry* 50 27–36. 24315837 10.1016/j.pnpbp.2013.11.014

[B84] Sun M. Choi E. Y. Magee D. J. Stets C. W. During M. J. Lin E. J. D. (2014). Metabolic effects of social isolation in Adult C57BL/6 Mice. *Int. Sch. Res. Notices* 2014:690950. 10.1155/2014/690950 27433503 PMC4897244

[B85] Tiihonen J. Kuikka J. Bergstrom K. Lepola U. Koponen H. Leinonen E. (1997). Dopamine reuptake site densities in patients with social phobia. *Am. J. Psychiatry* 154 239–242. 10.1176/ajp.154.2.239 9016274

[B86] Toth I. Neumann I. D. (2013). Animal models of social avoidance and social fear. *Cell Tissue Res.* 354 107–118. 10.1007/s00441-013-1636-4 23760888

[B87] Treadway M. T. Zald D. H. (2013). Parsing anhedonia: translational models of reward-processing deficits in psychopathology. *Curr. Dir. Psychol. Sci.* 22 244–249. 10.1177/0963721412474460 24748727 PMC3989147

[B88] Venzala E. Garcia-Garcia A. L. Elizalde N. Tordera R. M. (2013). Social vs. environmental stress models of depression from a behavioural and neurochemical approach. *Eur. Neuropsychopharmacol.* 23 697–708. 10.1016/j.euroneuro.2012.05.010 22743048

[B89] Watt M. J. Roberts C. L. Scholl J. L. Meyer D. L. Miiller L. C. Barr J. L. (2014). Decreased prefrontal cortex dopamine activity following adolescent social defeat in male rats: role of dopamine D_2_ receptors. *Psychopharmacology* 231 1627–1636. 10.1007/s00213-013-3353-9 24271009 PMC3969403

[B90] Wittchen H. U. Fuetsch M. Sonntag H. Müller N. Liebowitz M. (1999). Disability and quality of life in pure and comorbid social phobia–Findings from a controlled study. *Eur. Psychiatry* 14 118–131. 10.1016/s0924-9338(99)80729-9 10572336

[B91] Xu Y. Schneier F. Heimberg R. G. Princisvalle K. Liebowitz M. R. Wang S. (2012). Gender differences in social anxiety disorder: results from the national epidemiologic sample on alcohol and related conditions. *J. Anxiety Disord.* 26 12–19. 10.1016/j.janxdis.2011.08.006 21903358

[B92] Zhang F. Yuan S. Shao F. Wang W. (2016). Adolescent social defeat induced alterations in social behavior and cognitive flexibility in adult mice: effects of developmental stage and social condition. *Front. Behav. Neurosci.* 10:149. 10.3389/fnbeh.2016.00149 27489540 PMC4951521

